# Cellular Anti-Inflammatory and Antioxidant Activities of Bamboo *Sasa albomarginata* Leaf Extract and Its Constituent Coumaric Acid Methyl Ester

**DOI:** 10.1155/2022/8454865

**Published:** 2022-10-25

**Authors:** Shiori Kojima, Masatoshi Hakamata, Toshimichi Asanuma, Rie Suzuki, Jun-ichi Tsuruda, Takeshi Nonoyama, Yinzhi Lin, Hitomi Fukatsu, Naoki Koide, Kazuo Umezawa

**Affiliations:** ^1^Fukuyu Medical Institute, Fukuyu Medical Corporation, Nisshin, Aichi 470-0103, Japan; ^2^Department of Molecular Target Medicine, Aichi Medical University, Nagakute 480-1195, Japan; ^3^Food Science and Technology Section, Industrial Research Institute of Shizuoka Prefecture, Shizuoka 421-1298, Japan; ^4^Fukuyu Hospital, Fukuyu Medical Corporation, Nisshin, Aichi 470-0103, Japan; ^5^Department of Microbiology and Immunology, Aichi Medical University, Nagakute 480-1195, Japan

## Abstract

**Background:**

Hot water extract of *Sasa albomarginata* (Kumazasa) leaves is commercially available and used as a dietary supplement or skincare cream. The extract possesses anti-inflammatory activity on the mouse atopic dermatitis model. To elucidate the mechanism of in vivo activity, we have studied the cellular anti-inflammatory and antioxidant activities of the extract and its constituents.

**Methods:**

Secretion of mouse and human IL-6 was measured by ELISA. ROS production was measured by a fluorescent reagent. Ultrahigh performance liquid chromatography (UHPLC)/MS was used for the ingredient analysis.

**Results:**

The *Sasa albomarginata* extract inhibited inflammatory mediators such as LPS-induced NO, IL-6, and ROS production in mouse monocyte leukemia RAW264.7 cells. It also inhibited iNOS, IL-6, and IL-1*β* expressions. Moreover, it inhibited LPS-induced IL-6 expression and production in human monocyte leukemia THP-1 cells differentiated into macrophages. The HPLC analysis of the extract revealed the existence of coumaric acid, ferulic acid, and coumaric acid methyl ester. Coumaric acid methyl ester but not coumaric acid or ferulic acid inhibited LPS-induced NO, IL-6, and ROS production in RAW264.7 cells and IL-6 production in differentiated THP-1 cells.

**Conclusion:**

The hot water extract of *Sasa albomarginata* leaves and one of its constituents possess cellular anti-inflammatory and antioxidant activities.

## 1. Introduction

The moisture cream is widely used to protect skin from dryness and cracking. To provide moisture to skin, glycerin, mineral oil, hyaluronic acid, and so on are used. If anti-inflammatory compounds are added to these constituents, the cream should be more effective to protect the skin even from cracking and inflammation. Especially, pressure ulcers are often a serious problem in most nursing homes. For this purpose, natural materials such as edible plant extracts would be more advantageous than chemicals because of ease of development. *Sasa albomarginata* is a bamboo called Kumazasa often found in Hokkaido, Japan, and Sichuan, China. Latin names of Kumazasa include *Sasa albomarginata*, *Sasa veitchii*, and *Sasa senanensis*. The leaves are well known as food for pandas. The extract was reported to possess antibacterial activity. The organic solvent extract of *Sasa albomarginata* leaves was reported to show antimicrobial activity against bacteria, fungi, and yeast. Both acidic and phenolic fractions of the extract showed antimicrobial activity [1]. Cytomegalovirus is one of the herpesviruses. The hot water-soluble fraction of *Sasa albomarginata* inhibited the cellular infection of human cytomegalovirus [2]. They isolated five compounds from the extract, and among them, tricin showed antiviral activity in a plaque assay with a human embryonic fibroblast cell line. The antiviral activity of hot water extract was also shown in the plaque assay for the pseudorabies virus, a kind of herpesvirus [3]. Antitumor activity was reported for an alkaline extract of *Sasa senanensis* known as Sasa Health [4]. The extract was added to the drinking water and inhibited both the onset and growth of spontaneous mammary tumors in a high mammary tumor strain of SHN virgin mice. Sasa Health also inhibited spontaneous mammary tumorigenesis in another mouse model with human breast cancer [5]. Oral administration of Sasa Health delayed the development of tumors. It also inhibited mammary duct branching and side bud development with reduced angiogenesis. Kumazasa leaves are known to possess anti-inflammatory activity. Oral administration of a hot water extract of *Sasa albomarginata* leaves significantly reduced the incidence of stress-, ethanol-induced and indomethacin-induced gastric ulcers in rats [6]. The extract also inhibited a release of histamine from rat mast cells [6]. The methanol extract of *Sasa veitchii* did not inhibit NF-*κ*B activation but inhibited cyclooxygenase activity [7]. Moreover, the hot water extract of *Sasa albomarginata* was reported to ameliorate a mouse model of atopic dermatitis by topical administration [8]. The study employed DS-*Nh* mice in which atopic dermatitislike lesions were spontaneously developed with an increase of serum IgE. The extract ameliorated the skin inflammation score and decreased serum IgE. Since the hot water extract of *Sasa albomarginata* leaves possesses anti-inflammatory activity on skin inflammation, skin cream containing the extract has been developed. Meanwhile, coumaric acid, ferulic acid, and caffeic acid were found to exist in the steam or hot water extract of *Sasa albomarginata* leaves [9]. These three compounds possess related cinnamic acid structures [10]. In the present research, we first studied the cellular anti-inflammatory and antioxidant activities on hot water extract of *Sasa albomarginata* leaves. Then, we tried to find the chemicals contained in *Sasa albomarginata* extract that possess anti-inflammatory and antioxidant activities.

## 2. Materials and Methods

### 2.1. Materials

The hot water extract of *Sasa albomarginata* leaves (Kumazasa extract) was purchased from Toyo Ink Co., Ltd. (Tokyo, Japan). *p*-Coumaric acid, caffeic acid, and ferulic acid were purchased from Sigma-Aldrich (St. Louis, MO). Coumaric acid methyl ester was purchased from Tokyo Chemical Industry (Tokyo, Japan).

### 2.2. Cell Culture

Mouse monocytic leukemia RAW264.7 cells (Riken Bioresource Center, Tsukuba, Japan) were cultured in RPMI 1640 medium supplemented with 10% (v/v) fetal bovine serum and 1% (v/v) penicillin/streptomycin at 37°C in a humidified incubator with 5% CO_2_. Human monocytic leukemia THP-1 cells (Riken Bioresource Center, Tsukuba, Japan) were cultured in RPMI 1640 medium supplemented with 10% (v/v) fetal bovine serum and 1% (v/v) penicillin/streptomycin at 37°C in a humidified incubator with 5% CO_2_. THP-1 cells were seeded at 1.5 × 10^4^ cells/well onto 96-well plates and differentiated into macrophagelike cells with 0.1 *µ*g/ml phorbol 12-myristate 13-acetate (PMA; Sigma-Aldrich, St. Louis, MO) for 72 h.

### 2.3. Cell Viability

RAW264.7 cells at a density of 3 × 10^5^ cells/ml were seeded onto 96-well plates and incubated for 1 h. Differentiated THP-1 cells were washed with PBS and fresh medium and incubated for 24 h or 4 h. The test chemicals were added to each well and further incubated for 24 h. Next, MTT solution was added to each well and incubated for 2 h. Then, the supernatant was replaced by 100 *µ*l DMSO and pipetted to dissolve formazan crystals. Absorbance was determined on a microplate reader (Bio-Rad Laboratories, Inc., Hercules, CA) at 570 nm.

### 2.4. Nitric Monoxide Production

RAW264.7 cells at a density of 3 × 10^5^ cells/ml were seeded onto 96-well plates and incubated for 1 h. The test chemicals were added to each well and further incubated for 1 h and then exposed to 100 ng/ml LPS for 24 h at 37°C. The supernatant was harvested and then 50 *μ*l aliquots were mixed with an equal volume of Griess reagent in a 96-well plate. Absorbance was then determined on a microplate reader (Bio-Rad, Hercules, CA) at 570 nm.

### 2.5. Measurement of Mouse IL-6 Production

RAW264.7 cells at a density of 3 × 10^5^ cells/ml were seeded onto 96-well plates and incubated for 1 h. The test compound solutions at different concentrations were added to each well and further incubated for 1 h and then exposed to 100 ng/ml LPS for 24 h at 37°C. IL-6 concentration in culture supernatants was measured using Mouse IL-6 ELISA kit (R&D Systems, Minneapolis, MN). Briefly, the assay diluent and the supernatants were added to the well and incubated at room temperature for 2 h. Then, the plate was washed 5 times, and Mouse IL-6 conjugate was added to the well for 2 h. The plates were washed 5 times and freshly mixed substrate solution was added to each well and incubated for 30 min at room temperature in the dark. Then, diluted hydrochloric acid was added to stop the reaction. The absorbance was measured at 450 nm by the microplate reader (Bio-Rad, Hercules, CA).

### 2.6. Measurement of Human IL-6 Production

THP-1 cells at a density of 1.5 × 10^5^ cells/ml were seeded onto 96-well plates and differentiated into macrophagelike cells using PMA. Differentiated THP-1 cells were washed with PBS and fresh medium and incubated for 24 h. The test chemical solutions were added to each well and further incubated for 1 h and then exposed to 100 ng/ml LPS for 24 h at 37°C. IL-6 concentration in culture supernatants was measured using Human IL-6 ELISA kit (R&D Systems, Minneapolis, MN). Briefly, the assay diluent and the supernatants were added to the well and incubated at room temperature for 2 h. Then, the plate was washed 4 times, Human IL-6 conjugate was added to the well and incubated at room temperature for 2 h. The plates were washed 4 times, and freshly mixed substrate solution was added to each well and incubated for 20 min at room temperature in the dark. Then, 2 N Sulfuric Acid was added to stop the reaction. The absorbance was measured at 450 nm by the microplate reader (Bio-Rad, Hercules, CA).

### 2.7. Measurement of Reactive Oxygen Species (ROS) Production

RAW264.7 cells at a density of 3 × 10^5^ cells/ml were seeded onto 96-well plates or 24-well plates incubated overnight. The test compound solutions were added to each well and further incubated for 1 h and then exposed to 100 ng/ml LPS for 24 h at 37°C. The production of ROS was determined using DCFH-DA (Sigma, St. Louis, MO), an oxidant-sensitive fluorescent probe. The medium was removed from each well, and the cells were washed with Ca^2+^, Mg^2+^-free PBS (PBS^−^) twice. After being incubated for 30 min with 20 *µ*M DCFH-DA, the supernatant was removed. Cells were then washed with PBS^−^ twice and added by 200 *µ*l PBS^−^. Thereafter, the fluorescence was read with excitation at 485 nm and emission at 535 nm on a fluorescence plate reader, and the cells were visualized by a fluorescence microscope (BZ-X800; Keyence, Osaka, Japan).

### 2.8. Measurement of the DPPH-Radical Scavenging Ratio

The 2,2-diphenyl-1-picrylhydrazyl (DPPH) scavenging effect was measured using DPPH Antioxidant Assay kit (Dojindo, Kumamoto, Japan). Briefly, sample solution (20 *µ*l) was added to each well in 96-well microplate, followed by 80 *µ*L assay buffer and 100 *µ*l DPPH working solution. Then, the plate was incubated for 30 min at room temperature in the dark. The absorbance was measured at 517 nm by the microplate reader.

### 2.9. RNA Isolation and Real-Time PCR

RAW264.7 cells were seeded onto 60 mm dishes and were incubated for 24 h. The test compound solutions at different concentrations were added to each dish and further incubated for 1 h and then exposed to 100 ng/ml LPS for 4 h at 37°C. Next, total RNA was extracted from the culture cells using TRIzol reagent (Thermo Fisher Scientific, Inc., Waltham, MA, USA) and reverse transcribed with High-Capacity cDNA Reverse Transcription kit (Thermo Fisher Scientific, Inc., Waltham, MA, USA). For real-time PCR, the prepared cDNA was added to 14 *µ*l of KOD FX Neo PCR Buffer and dNTPs (TOYOBO CO., LTD, Osaka, Japan), the respective primer pairs for each gene then amplified in triplicate at 94°C for 10 sec, 94°C for 10 sec, 60°C for 10 sec, 70°C for 20 sec, 40 cycles using QuantStudio 3 system (Applied Biosystems). GAPDH and *β*-actin were used as an internal control. The relative mRNA expression for various genes were calculated by using the comparative ΔΔCt method. The following primer pairs were used: IL-1*β*, 5′-CGTGGACCTTCCAGGATGAG-3′ (forward) and 5′-GGAGCCTGTAGTGCAGCTGTC-3′ (reverse); mouse IL-6, 5′-ACCACGGCCTTCCCTACT TC-3′ (forward) and 5′-CACAACTCTTTTCTCATTTCCACG-3′ (reverse); iNOS, 5′-TGCACCACCAACTGCTTAG-3′ (forward) and 5′-TCTCTGCCTATCCGTCTCGTC-3′ (reverse), human IL-6, 5′-AGACAGCCACTCACCTCTTCAG-3′ (forward) and 5′-TTCTGCCAGTGCCTCTTTGCTG-3′ (reverse); GAPDH, 5′-TGCACCACCAACTGCTTAG-3′ (forward) and 5′-GATGCAGGGATGATGTTC-3′ (reverse) and *β*-actin, 5′-CTTCTACAATGA GCTGCGTG-3′ (forward) and 5′-TCATGAGGTAGTCAGTCAGG-3′ (reverse).

### 2.10. Ultrahigh Performance Liquid Chromatography (UHPLC) Analysis

The UHPLC analysis was performed on a Waters Acquity UPLC system (Waters Corp., Milford, MA) to determine the amount of coumaric acid and ferulic acid. An Acquity BEH C18 column (2.1 mm × 100 mm, 1.7 *µ*m; Waters Corp.) was used at 40°C for the separation. The mobile phase consisted of water with 0.1% formic acid (Solvent A) and acetonitrile with 0.1% formic acid (Solvent B). The mobile phase flow rate was 0.2 ml/min. The gradient program was as follows: 10% Solvent B at 0–0.5 min; 10–50% at 0.5–8 min; 50–60% at 8–8.1 min; 60% at 8.1–9 min; 60–10% at 9–9.1 min; 10% at 9.1–10 min. The total run was 10 min. The autosampler was set at 15°C and the injection volume of each sample was 2 *µ*l. The signals were detected at 325 nm.

### 2.11. UHPLC-MS Analysis

To quantify the concentration of coumaric acid methyl ester, mass spectrometry was performed on an LCT premier XE (Waters Corp.). The separations were performed on the same UPLC system, column, temperature of the autosampler, injection volume of samples, and mobile phase at UHPLC analysis. The column oven was maintained at 30°C. The mobile phase gradient condition was as follows: 5% Solvent B at 0–0.5 min; 5–80% at 0.5–8 min; 80% at 8-9 min; 80-5% at 9-9.1 min; 5% at 9.1–10 min. The total run was 10 min. The mass spectrometer analysis was operated in the electrospray ionization negative mode. MS source conditions were set as follows: capillary voltage, 2.8 kV; source temperature, 120°C; desolvation temperature, 400°C; desolvation gas flow, 800 l/h; cone gas flow, 50 l/h; V mode.

### 2.12. Statistical Analysis

All results were shown as mean ± standard deviation (S.D.). Student's *t*-test was used to analyze differences between two groups. The differences over two groups were detected by one-way analysis of variance (ANOVA) followed by Dunnett's posthoc test.

## 3. Results

### 3.1. Inhibition of Inflammatory Mediator and ROS Production by *Sasa albomarginata* Extract in RAW264.7 Cells

**Figure 1:**
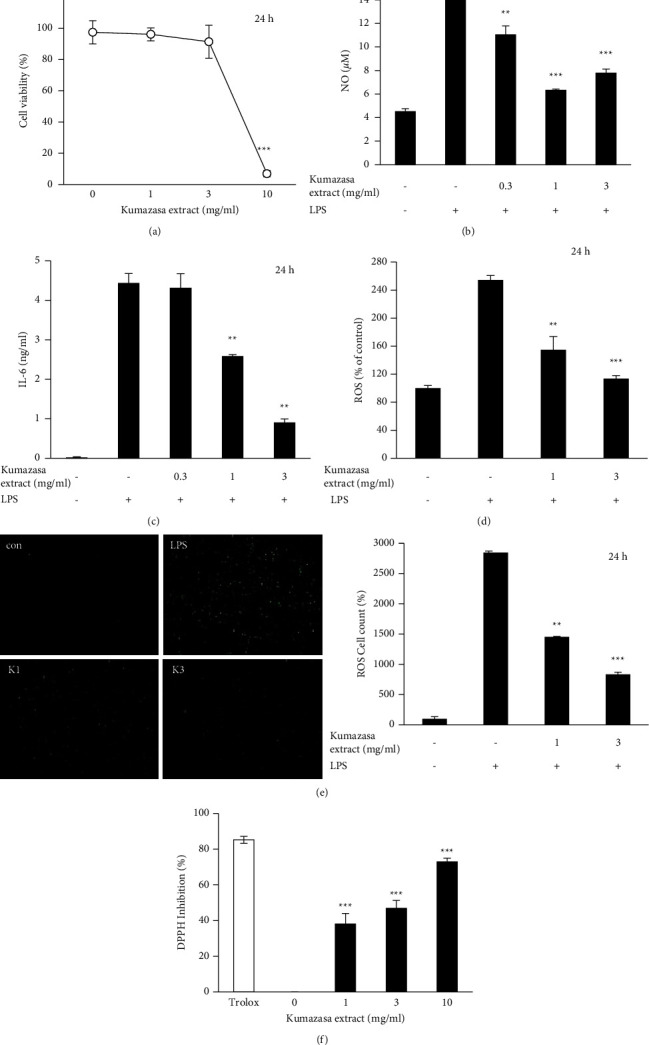
Inhibition of inflammatory mediator and ROS production by *Sasa albomarginata* extract in RAW264.7 cells. (a) Effect on cell viability. (b) Inhibition of NO production. (c) Inhibition of IL-6 production. (d), (e) Inhibition of LPS-induced ROS production. ROS was measured by the fluorescent intensity (d) or counting the fluorescent cells (e). The cells were stimulated with 100 ng/ml LPS with or without the extract. (f) In vitro antioxidant activity. In vitro antioxidant activity of *Sasa albomarginata* extract was evaluated by the DPPH radical-scavenging assay. Trolox concentration used was 80 *µ*g/ml.

The extract was dissolved directly in the medium. *Sasa albomarginata* extract did not show cytotoxicity below 3 mg/ml in RAW264.7 cells (Figure 1(a)). The extract inhibited LPS-induced NO production (Figure 1(b)) and IL-6 secretion (Figure 1(c)) at 3 mg/ml or lower concentrations. In Figure 1(b), a high concentration of the extract might influence the colorimetric assay. LPS induced ROS production in RAW264.7 cells, and the *Sasa albomarginata* extract inhibited ROS production at 1 and 3 mg/ml (Figures 1(d) and 1(e)). The fluorescence was measured directly (Figure 1(d)) or by counting the fluorescent cells (Figure 1(e)). The extract showed in vitro antioxidant activity in the assay using DPPH (Figure 1(f)).

### 3.2. Inhibition of iNOS, IL-6 and IL-1*β* Expressions by *Sasa albomarginata* Extract in RAW264.7 Cells

**Figure 2:**
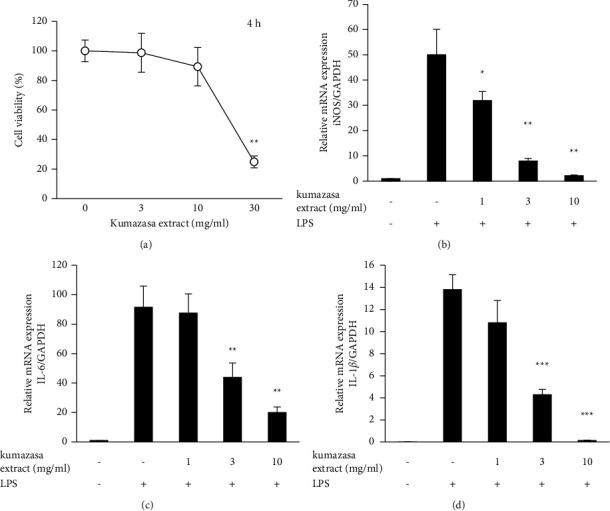
Inhibition of iNOS, IL-6 and IL-1*β* expressions by *Sasa albomarginata* extract in RAW264.7 cells. (a) Effect on cell viability. (b) Inhibition of iNOS expression. (c) Inhibition of IL-6 expression. (d) Inhibition of IL-1*β* expression. The cells were stimulated with 100 ng/ml LPS with or without the extract.

NO is produced by inducible NO synthase (iNOS). We have studied the effect of *Sasa albomarginata* extract on LPS-induced iNOS, IL-6, and IL-1*β* expressions using real-time PCR. The incubation time was 4 h, and the extract was not toxic below 10 mg/ml (Figure 2(a)). It inhibited iNOS (Figure 2(b)), IL-6 (Figure 2(c)) and IL-1*β* (Figure 2(d)) expressions at the nontoxic concentrations.

### 3.3. Inhibition of IL-6 Production and Expression by *Sasa albomarginata* Extract in Differentiated THP-1 Cells

**Figure 3:**
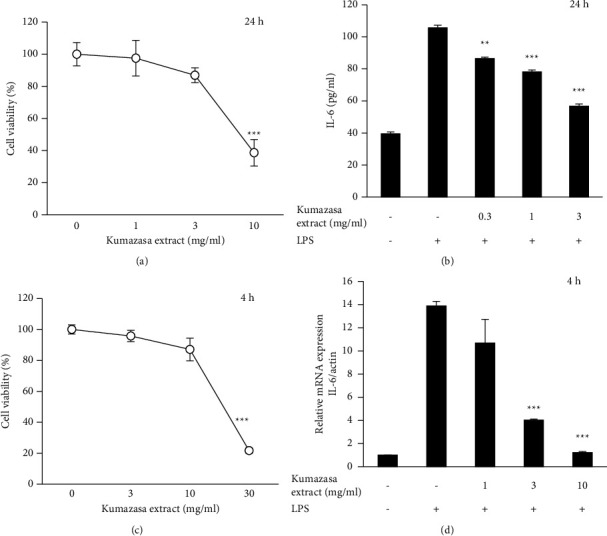
Inhibition of IL-6 production and expression by *Sasa albomarginata* extract in differentiated THP-1 cells. (a) Effect on cell viability in 24 h. (b) Inhibition of IL-6 production. (c) Effect on cell viability in 4 h. (d) Inhibition of IL-6 expression. The cells were stimulated with 100 ng/ml LPS with or without the extract.

THP-1 cells are human monocytic leukemia cells. This cell line can be differentiated into macrophage-like cells by incubation with PMA. Since the cells were treated with PMA, they are difficult to produce NO. However, LPS can activate IL-6 production. The *Sasa albomarginata* extract was not prominently toxic at 3 mg/ml (Figure 3(a)) in THP-1 cells. It also inhibited the LPS-induced IL-6 secretion in THP-1 cells at nontoxic concentrations (Figure 3(b)). We have also studied the effect of *Sasa albomarginata* extract on IL-6 expression using real-time PCR. The incubation time was 4 h, and the extract was not prominently toxic even at 10 mg/ml (Figure 3(c)). It also inhibited the LPS-induced IL-6 expression in THP-1 cells at nontoxic concentrations (Figure 3(d)).

### 3.4. HPLC Analysis of *Sasa albomarginata* Extract

**Figure 4:**
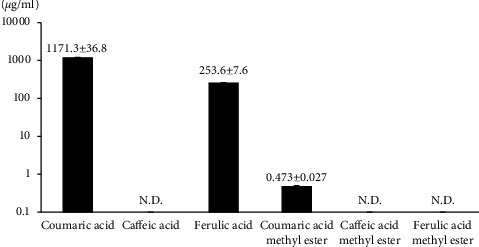
HPLC analysis of *Sasa albomarginata* water extract ingradients. Values are mean ± S.D. of 3 determinations. N.D., not detected.

Since coumaric acid, caffeic acid, and ferulic acid were reported to exist in the extract, we measured the content of these acids. As a result, large amounts of coumaric acid and ferulic acid but not caffeic acid were found in the extract. Then, we analyzed the cellular anti-inflammatory activity of these acids by LPS-induced NO production, but they showed no activity. Since acidic compounds may not pass though the cell membrane, we tested the anti-inflammatory activity of each ester. These methyl esters inhibited NO production. Then, we measured the content of these esters by HPLC. We found the existence of coumaric acid methyl ester as shown in Figure 4.

### 3.5. Inhibition of Inflammatory Mediator and ROS Production by Coumaric Acid Methyl Ester in RAW264.7 Cells

**Figure 5:**
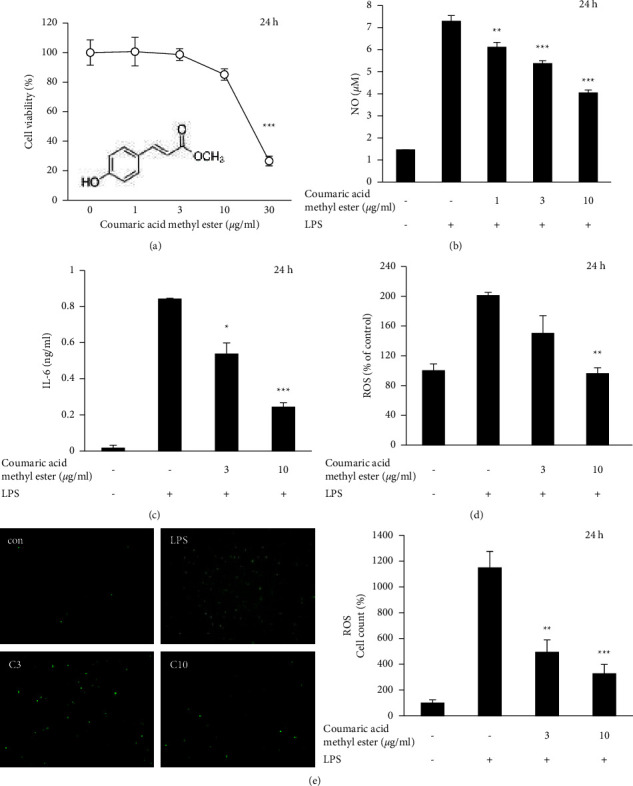
Inhibition of inflammatory mediator and ROS production by coumaric acid methyl ester in RAW264.7 cells. (a) Effect on cell viability. (b) Inhibition of NO production. (c) Inhibition of IL-6 production. (d, e) Inhibition of LPS-induced ROS production. ROS was measured by the fluorescent intensity (d) or counting the fluorescent cells (e). The cells were stimulated with 100 ng/ml LPS with or without the extract.

Coumaric acid methyl ester did not show cytotoxicity below 10 *μ*g/ml in RAW264.7 cells (Figure 5(a)). Coumaric acid methyl ester inhibited LPS-induced NO production (Figure 5(b)) and IL-6 secretion (Figure 5(c)) at 10 *μ*g/ml or lower concentrations. Coumaric acid methyl ester also inhibited the LPS-induced ROS production at 3 and 10 *μ*g/ml (Figures 5(d) and 5(e)). Fluorescence was measured directly (Figure 5(d)) or by counting the fluorescent cells (Figure 5(e)). Unlike the *Sasa albomarginata* extract (Figure 1(f)), the chemical did not show antioxidant activity in the same assay system.

### 3.6. Inhibition of IL-6 Production by Coumaric Acid Methyl Ester in Differentiated THP-1 Cells

**Figure 6:**
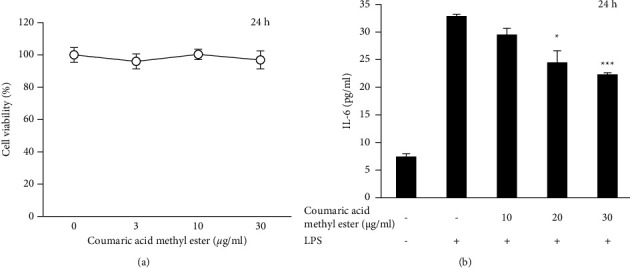
Inhibition of IL-6 production by coumaric acid methyl ester in differentiated THP-1 cells. (a) Effect on viability. (b) Inhibition of IL-6 production. The cells were stimulated with 100 ng/ml LPS with or without the chemical.

Coumaric acid methyl ester did not show cytotoxicity even at 30 *μ*g/ml (Figure 6(a)). This compound inhibited the LPS-induced IL-6 secretion in THP-1 cells at 20 and 30 *μ*g/ml (Figure 6(b)).

## 4. Discussion

Hot water extract of Kumazasa (*Sasa albomarginata*) inhibited LPS-induced cellular inflammation. The extract was found to contain comparatively large amounts of coumaric acid and ferulic acid, although they showed no anti-inflammatory activity. However, the methyl ester of coumaric acid contained in the extract showed cellular anti-inflammatory activity. Both coumaric acid and coumaric acid methyl ester contained in *Lavatera trimestris* flowers were reported to show in vitro radical scavenging activity [11]. Methanolic extracts from long English cucumber leaves were fractionated and tested for their antifungal activity against *Cladosporium cucumerinum*. After isolation and purification, the active principle was found to be coumaric acid methyl ester [12]. Coumaric acid methyl ester is one of the bioactive components of the plant *Costus speciosus*. This plant is traditionally used in Asia to treat catarrhal fevers and skin diseases. The coumaric acid methyl ester was found to inhibit migration and tube formation in human umbilical vein endothelial cells (HUVEC) possibly via inhibition of the VEGF-VEGFR2 pathway [13]. It also inhibited physiological and tumor angiogenesis in zebrafish [13]. Caffeic acid and ferulic acid structures are close to that of coumaric acid. Caffeic acid methyl ester but not caffeic acid was reported to show cellular and in vivo anti-inflammatory activity [14]. Therefore, it may be possible that the *Sasa albomarginata* extract and coumaric acid methyl ester would show in vivo anti-inflammatory activity. Coumaric acid methyl ester showed antioxidant activity in our study. Ferulic acid methyl ester also shows antioxidant activity, and the mechanism has been studied. Using ethyl linoleate as the oxidation substrate, the antioxidation reaction of ferulic acid methyl ester produced a dimer of ferulic acid methyl ester having a dihydrobenzofuran moiety [15]. A para-hydroxy group is necessary for the formation of dihydrobenzofuran moiety in this reaction. Since coumaric acid has a para-hydroxy group, the reported mechanism may be applicable to the anti-oxidant reaction of this compound. A large amount of coumaric acid and ferulic acid exists in the water extract of Kumazasa leaves. But they did not inhibit LPS-induced NO production at the non-toxic concentrations. Possibly, these compounds are too polar to penetrate the cell membrane. On the other hand, we have found coumaric acid methyl ester contained in the extract (Figure 4), inhibited LPS-downstream signaling as shown in Figures 5 and 6. It may contribute to the anti-inflammatory activity of the extract. However, since the content is comparatively little, the contribution is likely to be limited. It is possible that coumaric acid and ferulic acid are endogenously esterified by lipase [16]. Song et al. has studied permeability and antimelanogenic effects of coumaric acid and coumaric acid methyl ester [17]. In an ex vivo skin permeation assay, topically applied coumaric acid rather than the methyl ester in the cream could more effectively diffuse into the aqueous medium underneath the skin. Thus, in our case, coumaric acid and ferulic acid themselves are likely to show anti-inflammatory activity in vivo with or without esterification. This time we employed hot water extract of *Sasa albomarginata* leaves because of the reported in vivo anti-inflammatory activity. However, it is likely that alcoholic extraction may provide a more potent extract because of the effective isolation of esterified compounds.

The plant extract showed potent antioxidant activity in vitro, while coumaric acid methyl ester did not inhibit in vitro. It is possible that the chemical modifies NF-*κ*B related signaling to inhibit the production of ROS [18]. Plant extracts are generally considered to be safe and they often show anti-inflammatory or antimicrobial activity. *Launaea sarmentosa* extract inhibited LPS-induced cellular inflammation via suppression of NF-*κ*B/MAPK signaling with Nrf2 activation [19]. *Cannabis* extract and its ingredient cannabidiol inhibited the productions of cellular mediators of skin inflammation [20]. *Smilax guianensis* extract prevented LPS-induced cellular inflammation by inhibiting the NF-*κ*B pathway in RAW264.7 cells [21]. Methanol extract from *Saussurea involucrata* inhibited the LPS-stimulated inflammatory responses also in cultured RAW264.7 cells [22]. *Azadirachta indica* bark extract restricted coronaviral infection and replication model [23]. Kumaizasa (*Sasa senanensis*) leaf extract was reported to inhibit LPS-induced IL-1*β* and TNF-*α* expression in macrophages but increased IL-8 production in HEK293 cells [24]. Alkaline extract of *Sasa* sp. inhibited IL-1*β*-induced prostaglandin E2 in human gingival fibroblasts [25]. Kim et al. prepared a cellular inflammatory bowel disease (IBD) model with coculture of human intestinal epithelial Caco-2 cells and RAW264.7 cells [26]. *Sasa quelpaertensis* leaf extract suppressed LPS-induced inflammatory mediator expressions in this system.

## 5. Conclusion

The hot water extract of *Sasa albomarginata* leaves inhibited LPS-induced inflammatory responses and ROS production at the nontoxic concentrations in macrophage-like cells. The extract was shown to contain coumaric acid, ferulic acid, and small amount of coumaric acid methyl ester. Since coumaric acid methyl ester showed anti-inflammatory and antioxidant activities, the active principles may include coumaric acid and related compounds.

## Data Availability

The data used to support the findings of this study are included within the article.
